# Supplementary material for: Predicting visual function by interpreting a neuronal wiring diagram
Source: Nature. 2024 Oct 2;634(8032):113–23. doi: 10.1038/s41586-024-07953-5 (PMC11446822; doi:10.1038/s41586-024-07953-5)
Supplement: Supplementary file 6 — CRF and ERF predictions for individual TmY4 and TmY9 cells. Analogous to Supplementary Data 3, but for TmY target types. Shown are the top four monosynaptic pathways, the strongest pathway passing through each of the top ten intermediary types (ranking from Extended Data Fig. 7), and the trisynaptic pathway Tm1–TmY–Dm3–TmY (see the section entitled Prediction of spatial normalization). [file 41586_2024_7953_MOESM6_ESM.zip › DataS4/TmY9qΓèÑ/720575940623058579.pdf]

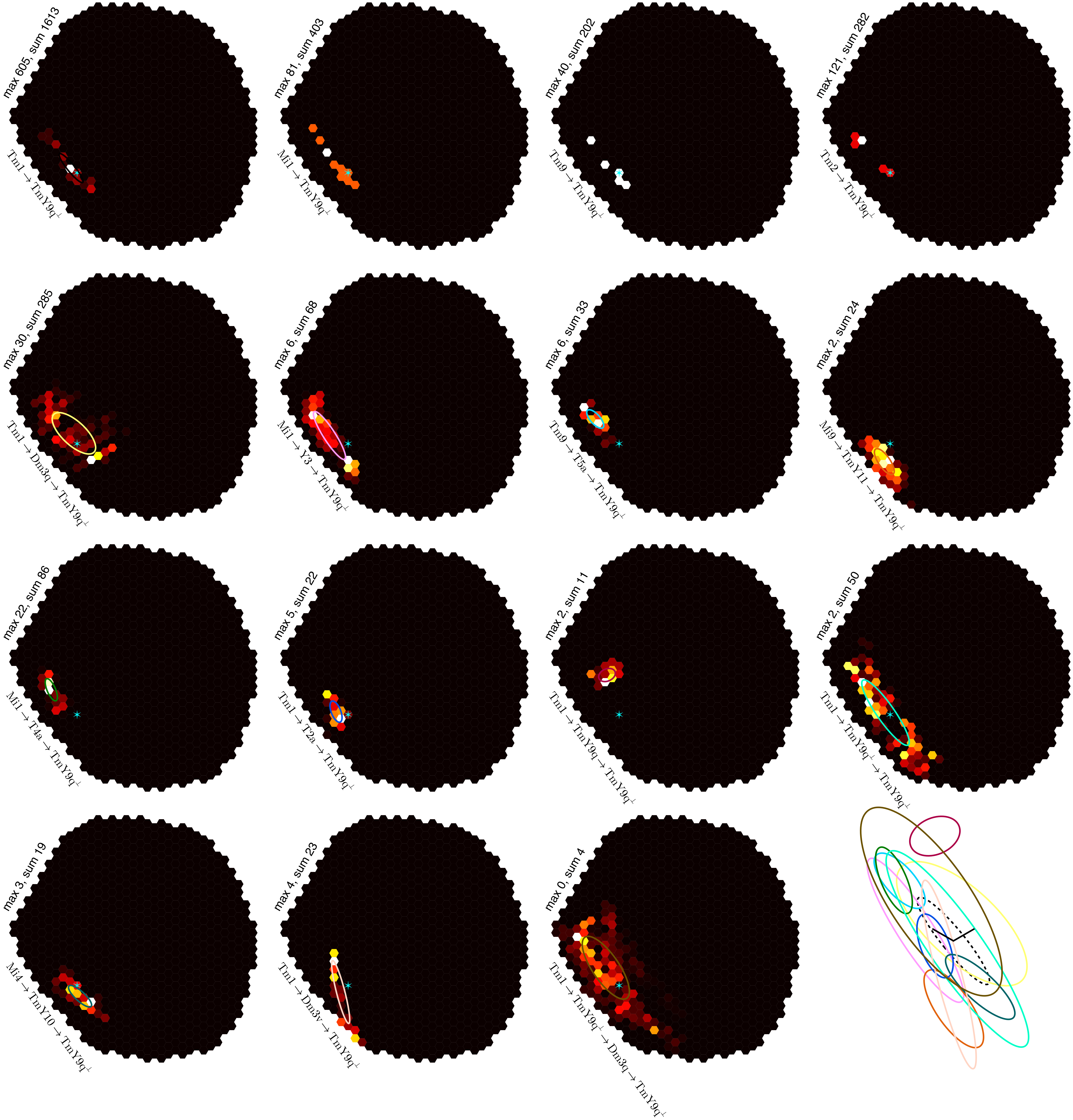
max 605, sum 1613
Tm1 → TmY9q⊥
max 81, sum 403
Mi1 → TmY9q⊥
max 40, sum 202
Tm9 → TmY9q⊥
max 121, sum 282
Tm2 → TmY9q⊥
max 30, sum 285
Tm1 → Dm3q → TmY9q⊥
max 6, sum 68
Mi1 → Y3 → TmY9q⊥
max 6, sum 33
Tm9 → T5a → TmY9q⊥
max 2, sum 24
Mi9 → TmY11 → TmY9q⊥
max 22, sum 86
Mi1 → T4a → TmY9q⊥
max 5, sum 22
Tm1 → T2a → TmY9q⊥
max 2, sum 11
Tm1 → TmY9q → TmY9q⊥
max 2, sum 50
Tm1 → TmY9q⊥ → TmY9q⊥
max 3, sum 19
Mi4 → TmY10 → TmY9q⊥
max 4, sum 23
Tm1 → Dm3v → TmY9q⊥
max 0, sum 4
Tm1 → TmY9q⊥ → Dm3q → TmY9q⊥
